# Low Structural Variation in the Host-Defense Peptide Repertoire of the Dwarf Clawed Frog *Hymenochirus boettgeri* (Pipidae)

**DOI:** 10.1371/journal.pone.0086339

**Published:** 2014-01-22

**Authors:** Severine Matthijs, Lumeng Ye, Benoit Stijlemans, Pierre Cornelis, Franky Bossuyt, Kim Roelants

**Affiliations:** 1 Amphibian Evolution Lab, Biology Department, Vrije Universiteit Brussel, Brussels, Belgium; 2 Department of Bioengineering Sciences, Research Group of Microbiology and Vlaams Instituut voor Biotechnologie, Vrije Universiteit Brussel, Brussels, Belgium; 3 Unit of Cellular and Molecular Immunology, Vrije Universiteit Brussel, Brussels, Belgium; 4 Laboratory of Myeloid Cell Immunology, Vlaams Instituut voor Biotechnologie, Brussels, Belgium; University of Utah, United States of America

## Abstract

The skin secretion of many amphibians contains peptides that are able to kill a broad range of microorganisms (antimicrobial peptides: AMPs) and potentially play a role in innate immune defense. Similar to the toxin arsenals of various animals, amphibian AMP repertoires typically show major structural variation, and previous studies have suggested that this may be the result of diversifying selection in adaptation to a diverse spectrum of pathogens. Here we report on transcriptome analyses that indicate a very different pattern in the dwarf clawed frog *H. boettgeri.* Our analyses reveal a diverse set of transcripts containing two to six tandem repeats, together encoding 14 distinct peptides. Five of these have recently been identified as AMPs, while three more are shown here to potently inhibit the growth of gram-negative bacteria, including multi-drug resistant strains of the medically important *Pseudomonas aeruginosa*. Although the number of predicted peptides is similar to the numbers of related AMPs in *Xenopus* and *Silurana* frog species, they show significantly lower structural variation. Selection analyses confirm that, in contrast to the AMPs of other amphibians, the *H. boettgeri* peptides did not evolve under diversifying selection. Instead, the low sequence variation among tandem repeats resulted from purifying selection, recent duplication and/or concerted gene evolution. Our study demonstrates that defense peptide repertoires of closely related taxa, after diverging from each other, may evolve under differential selective regimes, leading to contrasting patterns of structural diversity.

## Introduction

In situations of acute stress, like injury or a predator attack, many amphibians produce a skin secretion containing a complex mixture of peptides. Some of these peptides have been identified as structural analogues of evolutionary conserved vertebrate hormones, and have been shown to bind the same hormone receptors in a range of vertebrates. These hormone-like peptides (HLPs) have been hypothesized to provide passive defense against predation, by interfering with gastrointestinal processes upon ingestion [1], [2], [3]. Other peptides show potent cytolytic activity against a broad range of microorganisms, including bacteria, fungi and protozoan parasites. Most of these antimicrobial peptides (AMPs) are small to medium-sized (<30 amino acids), have an alternated sequence of hydrophobic and polar/cationic residues, and are able to adopt an alpha-helical structure upon contact with cell membranes. The resulting amphipathic conformation, with hydrophobic and polar residues on opposite sides of the helical axis, allows the targeting of negatively charged phospholipid heads on the outer surface of microorganisms, followed by intrusion of the cell membrane, pore formation, and eventually, cell lysis [4], [5]. Because most amphibian AMPs are more effective against microorganisms than against most cells of multicellular eukaryotes, they are generally considered a component of the amphibian’s innate immune system (but see [6]). Some amphibian species may produce up to well over 100 different AMPs [7], [8], although in most taxa the number of peptides typically lies around 5–20 [9], [10]. However, regardless of the size of the peptide repertoire, AMPs of a single species typically show major variation in AA sequence. As a result, they are often classified in different peptide “families” upon sequence comparison [7], [11], [12]. In some cases, this variation has been found to be the result of diversifying selection [9], [10], [13], potentially in adaptation to a diverse spectrum of pathogenic microorganisms [9], [10]. Although high sequence variation characterizes the AMP repertoires of amphibians across a broad phylogenetic diversity, the universality of this pattern is unknown.

The potential of both AMPs and HLPs in medical application fields like disease modeling and drug design, has turned amphibian skin peptidomics into an active research field. Some of the most intensively screened amphibians belong to the frog family Pipidae, which is composed of five aquatic genera in tropical Africa (*Hymenochirus*, *Pseudhymenochirus*, *Silurana* and *Xenopus*) and South America (*Pipa*). One of the earliest investigated species was the African clawed frog *Xenopus laevis*. Besides the HLPs caerulein [14] (resembling the vertebrate hormones cholecystokinin and gastrin) and xenopsin [15] (resembling the hormones neurotensin and xenin), *X. laevis* produces a variety of AMPs, nowadays classified in four evolutionarily related, but structurally distinct families [13], [16]: (i) magainin [17], (ii) caerulein precursor fragment (CPF), (iii) peptide glycine-leucine-amide (PGLa) [18], and (iv) xenopsin precursor fragments (XPF). Subsequent studies have shown that other *Xenopus* species, as well as species of the sister genus *Silurana*, produce similarly diverse AMP repertoires, composed of homologous peptides of the same four families [16], [19], [20], [21], [22], [23], [24], [25], [26]. Recent phylogenomic analyses have confirmed that the genes encoding these AMP families arose from the hormone gene cholecystokinin (cck) and diverged from each other through a process of gene duplication and diversifying selection [13]. As previous studies remained mostly limited to *Silurana* and *Xenopus*, the timing of AMP diversification relative to the early pipid phylogeny, and resulting patterns of AMP variation in other genera, remain unknown.

A recent screening for AMPs in the skin secretion of the dwarf clawed frog species *Hymenochirus boettgeri* yielded five peptides, which seem distantly related to *Xenopus* and *Silurana* AMPs [27]. Unlike the latter however, the *H. boettgeri* peptides were classified in a single family and named hymenochirin-1B through hymenochirin-5B. Here we use transcriptome analyses to further uncover the host-defense repertoire of *H. boettgeri*. The obtained transcript sequences provide insights in the genetic mechanisms underlying AMP evolution and synthesis, and deliver additional sequence data to conduct phylogenetic and selection analyses. Besides characterizing several new AMPs with promising activity against multidrug-resistant bacteria, our analyses reveal an unusual pattern of AMP variation, and further elucidate the functional evolution of one of the most intensively investigated amphibian skin defense arsenals.

## Materials and Methods

### Ethics Statement

Six captive-bred adult male *H. boettgeri* were purchased from a local pet shop and immediately euthanized while avoiding unnecessary stress or pain. The frogs were first anesthetized by immersion in 0.5 g/L buffered MS-222 (Sigma-Aldrich) and then euthanized by decapitation and pithing of the brain and spinal canal. This procedure does not violate any European convention (European Convention for the protection of Vertebrate animals used for experimental and other scientific purposes; CETS #123), Belgian law (Art. 2.6 of the Belgian Law of May 4th 1995), or institutional regulation, and thus requires no approval by the Ethical Committee for Animal Research of the Vrije Universiteit Brussel. Various tissues were sampled from each individual. The supporting ARRIVE Guidelines checklist for this study is available as supporting information (Checklist S1).

### cDNA Library Synthesis from Breeding Gland

Male *H. boettgeri* have a specialized, sexually dimorphic, granular gland behind each front limb (postaxillary), termed “breeding gland” [28], which substantially enlarges in the breeding period. Breeding glands from two male *H. boettgeri* were sampled and stored overnight in RNA Later® (Qiagen) at 4°C. Total RNA was extracted from the breeding glands (combined for both males) using the TRI-reagent protocol (Sigma-Aldrich). The RNA extracts of the glands were sent to Advanced BiotekServices (San Diego, CA, U.S.A.) for construction of a standard cDNA library with mammalian expression vector pCMVEXP. The *E. coli* culture transformed with cDNA library plasmids was diluted with SOB medium and plated on LB/ampicillin plates. Colonies were randomly selected the next day. The CMV forward primer and TK pA reverse primer were used to amplify the inserts. The polymerase chain reaction (PCR) was performed using Taq DNA Polymerase (Fermentas) under the following conditions: 94°C for 4 minutes, then 25 cycles of 94°C for 40 s, 63°C for 1 min and 72°C for 1 min, and a final extension step of 2 min at 72°C. A total of 576 PCR products was purified (Wizard® SV 96 PCR Clean-Up System, Promega), amplified with the CMV forward primer using the BigDye Terminator Sequencing Kit v.3.1 and sequenced on an ABI Prism 3100 automated sequencer (Applied Biosystems). The resulting sequences were assembled into contiguous sequences (contigs) using the CodonCode Aligner software (CodonCode Corporation). These contigs were compared to the database of the National Center for Biotechnology Information (NCBI) using the Basic Local Alignment Search Tool (BLAST) to identify potential AMP encoding transcripts.

### cDNA Synthesis from Skin Extracts by RACE PCR

Sampled skin tissue fragments from four male *H. boettgeri* were stored overnight in RNA Later® (Qiagen) at 4°C. Total RNA was extracted from the skin tissue (combined for the four males) using the TRI-reagent protocol (Sigma-Aldrich). Five prime cDNA was synthesized using the SMARTer™ RACE cDNA Amplification Kit (Clontech). Rapid Amplification of cDNA Ends (RACE) was performed with FastStart High Fidelity Polymerase (Roche Diagnostics), the 3′ universal primer A mix (UPM) of the kit and one of two primers specifically designed to amplify pipid AMP gene transcripts. Primer design was based on an alignment of amphibian cck genes, *Xenopus* and *Silurana* AMP genes, and a hymenochirin transcript sequence precursor obtained from the breeding gland cDNA library. Amplification products obtained by these primers were expected to be of different lengths as one primer, 5′-AGTACATCCATCYNYNCNGAGCA-3′ was designed on the 5′UTR, while the second, 5′-GGATATTTGCCTGYNTRYTNYTTGC-3′, corresponds to part of the signal peptide-encoding sequence. The amplification products of primer A were purified by gel extraction (Qiagen Gel Extraction Kit) and of primer B by total PCR-product purification (Qiagen PCR Purification Kit). Ligation of the purified PCR product in the pGEM-T Easy vector was done overnight with T4 DNA ligase. Products from the ligation reaction were then used to transform TOP10 chemically competent cells, spread on LB Agar plates (with ampicillin, IPTG and X-Gal) and incubated overnight. White colonies were picked and used for PCR with the corresponding primers and FastStart Taq polymerase (Roche Diagnostics). Positive PCR products were purified (Wizard® SV 96 PCR Clean-Up System), amplified in a cycle sequencing PCR with our primers using the BigDye Terminator Sequencing Kit v.3.1 (Applied Biosystems) and sequenced on an ABI Prism 3100 automated sequencer (Applied Biosystems). The cDNA sequences encoding hymenochirins were deposited in GenBank under accession numbers KF878086 through KF878101.

### Structural and Functional Analyses of Peptides

The presence of N-terminal signal peptides was predicted using SignalP 4.0 [29]. Secondary structure of the predicted hymenochirins was predicted using a neural network algorithm as implemented on the PSIPRED Protein Structure Prediction Server (PSIPRED v3.0) [30]. Peptide regions predicted to have an alpha-helical structure with high confidence (confidence levels 5 to 9 in the PSIPRED output) were projected in helical wheel diagrams to visualize their amphipathic nature. Hydrophobic moments were calculated using the combined consensus scale implemented in HydroMCalc [31].

Four of the predicted peptides were synthesized using solid-phase technology and HPLC purified to more than 95% purity by CASLO Laboratory ApS (Lyngby, Denmark). They were delivered as lyophilized trifluoroacetate salts. The synthetic peptides were dissolved in 0.01% acetic acid/0.2% BSA to a stock concentration of 5.12 mM. Antimicrobial activity of the four peptides was evaluated by determining the minimum peptide concentration in a series of twofold dilutions at which no bacterial growth could be detected (minimum inhibitory concentration (MIC)), following the protocol of Wiegand et al. [32]. Target species included one gram-positive strain, *Staphylococcus aureus* (NCTC 8325), and three gram-negative strains; *Escherichia coli* (MG1655), *Pseudomonas aeruginosa* PAO1, and *P. aeruginosa* PA7. The multidrug-resistant *P. aeruginosa* PA7 strain, a non-respiratory clinical isolate from Argentina, was kindly provided by Dr. P.H. Roy and co-workers [33]. Bacterial cultures (5×10^5^ colony forming units/ml) were prepared in Müller-Hinton (MH) broth and transferred to serial peptide dilutions ranging from 512 or 256 µM down to 1 or 0.5 µM in 96-well polypropylene plates. For each series of peptide dilutions, both a positive control (bacterial culture without peptide, to verify normal bacterial growth), and a negative control (MH broth without bacterial culture to detect potential contamination) were added to the plate. After incubation at 37°C for 18–20 hours, growth of the cultures in each of the wells was checked by eye. Because the bacteria were incubated without shaking and the 96-well plates had a round bottom, any bacterial growth would appear as a pellet at the bottom of the well. The antibacterial activity against *E. coli* and *S. aureus* was tested in four replicates, the antibacterial activity against *P. aeruginosa* PAO1 and *P. aeruginosa* PA7 was tested in triplicate.

Hemolytic activity against human erythrocytes was examined by assessing the lowest peptide concentration (in the same series of twofold dilutions) causing at least 50% hemolysis (LC50). Heparinized blood was obtained from a healthy donor, diluted 1/100 in PBS medium, and added to polypropylene 96-well plates containing serial peptide dilutions to obtain total volumes of 100 µl. Blood with 10% Tween-20 was used as a positive control because this results in 100% hemolysis. The plates were incubated at 37°C for 1 hour and 30 minutes and centrifuged at 1400 rpm for 2 minutes to pellet intact red blood cells allowing visual observation of hemolysis. Supernatants were subsequently transferred to 96-well flat-bottom plates to allow spectrophotometry analysis (OD measured at 550 nm) using an EL_X_808 microplate reader (Bio-Tek Instruments. Inc.). The percentage of hemolysis for each peptide concentration was calculated as 100×(ODobs – OD0%)/(OD100%– OD0%), where ODobs is the OD measured for the peptide concentration, OD0% is the average OD in the absence of peptides (0% hemolysis), and OD100% is the average OD in the presence of 10% Tween-20 (100% hemolysis).

### Sequence Alignment and Phylogenetic Analyses

Preliminary alignments identified repeat sequences in all obtained transcript sequences (see *Results*). Assuming that these repeats probably arose by tandem exon duplication, we delineated successive repeats by comparing the transcript sequences with the known exons of *Silurana tropicalis* and *X. laevis* AMP genes. As several exon boundaries are shared by all known AMP genes and vertebrate cck hormone genes [13], [34], they may be conserved in *H. boettgeri* as well. Repeat sections were defined as a part of a repeat that corresponds to a single exon in the *S. tropicalis* and *X. laevis* genes. Next, to compare repeat organization among the different *H. boettgeri* transcripts and identify identical repeat sections, we created a transcript alignment by manually shuffling whole repeat sections rather than individual nucleotides, using the program MacClade 4.06 [35].

The presence of similar or identical repeat sections in otherwise dissimilar transcripts suggests that they may represent recombinant sequences (e.g. due to alternative splicing or concerted gene evolution), whose tandem repeat sections may represent conflicting evolutionary histories. To minimize the risk of spurious phylogenetic results due to recombination, we estimated evolutionary relationships among individual repeat sections rather than among the entire transcripts (i.e., treating repeat sections of the same transcript as separate “taxa”). The necessary alignments were created using the EINSI algorithm implemented in Mafft 6.704 [36]. We prepared two repeat section data sets, each matching a single exon of *S. tropicalis* and *X. laevis* AMP genes. Data set 1 includes 27 nonidentical anterior sections of transcript repeats, aligned with the corresponding exon 2 of *S. tropicalis* and *X. laevis* AMP genes, and of amphibian cck genes (to serve as outgroup sequences). Data set 2 includes 21 nonidentical posterior sections of transcript repeats. In this case, the corresponding exon 3 of *S. tropicalis* and *X. laevis* AMP genes was not included, because its high sequence divergence with respect to the hymenochirin repeats compromised reliable sequence alignment. Therefore, the phylogenetic trees obtained for the posterior repeat sections are unrooted and used only for selection analyses (see below).

Phylogenetic relationships were estimated by maximum likelihood bootstrapping and Bayesian phylogeny inference, using RAxML 7.0.4 (Randomized AXelerated Maximum Likelihood) [37] and MrBayes 3.1.2 [38], respectively. Bootstrap percentages for branch support were obtained by applying RAxML’s fast hill-climbing algorithm on 500 bootstrap replicates, using the general time-reversible (GTR) model of DNA substitution with gamma correction of among-site rate heterogeneity and estimation of the proportion of invariable sites (GTR+G+I). MrBayes analyses were executed under the same model (GTR+G+I). Two parallel runs of four incrementally heated (temperature parameter = 0.2) Markov chain Monte Carlo (MCMC) chains were performed, with a length of 10,000,000 generations, a sampling frequency of 1 per 1,000 generations, and a burn-in corresponding to the first 2,000,000 generations. Convergence of the parallel runs was confirmed by split frequency standard deviations (<0.01) and potential scale reduction factors (approximating 1.0) for all model parameters, as reported by MrBayes. Adequate posterior sampling was verified using Tracer 1.5 [39], by checking if the runs had reached effective sampling sizes >200 for all model parameters.

### Selection Analyses

We investigated whether diversifying (positive) or purifying (negative) selection affected the evolution of the predicted peptides and their cleavage sites using three different likelihood methods. All of them implement sitewise tests of selective pressures based on site-by-site estimation of the ratio of nonsynonymous over synonymous substitutions. The first method, random effects likelihood (REL), draws sitewise synonymous and nonsynonymous codon substitution rates from separate rate heterogeneity distributions, and implements an empirical Bayes approach to test for significant selection at any specific site [40]. The second method, fixed effects likelihood (FEL), uses a likelihood function to estimate substitution rates directly at each codon site without assuming that they fit an overall distribution of rate heterogeneity. The third method, single likelihood ancestor counting (SLAC), estimates site-specific substitution rates based on ancestral codon reconstruction and compares observed codon substitution numbers to expected ones. All methods were applied using the software package HyPhy as implemented on the DataMonkey webserver [41]. All analyses were based on a MG94 codon substitution model ‘crossed’ with a GTR nucleotide substitution model, and codon sites were identified as subject to diversifying or purifying selection at p-values <0.05 (FEL and SLAC) or Bayes factors >50 (REL). Separate analyses were conducted on data sets 1 (anterior repeat sections) and 2 (posterior repeat sections), using the respective Bayesian consensus phylograms as input trees. In addition, to accommodate phylogenetic uncertainty, all analyses were replicated for both data sets using 20 trees randomly sampled from the respective posterior tree sets, as created by MrBayes.

## Results

### Hymenochirin Transcript Diversity

**Figure 1:**
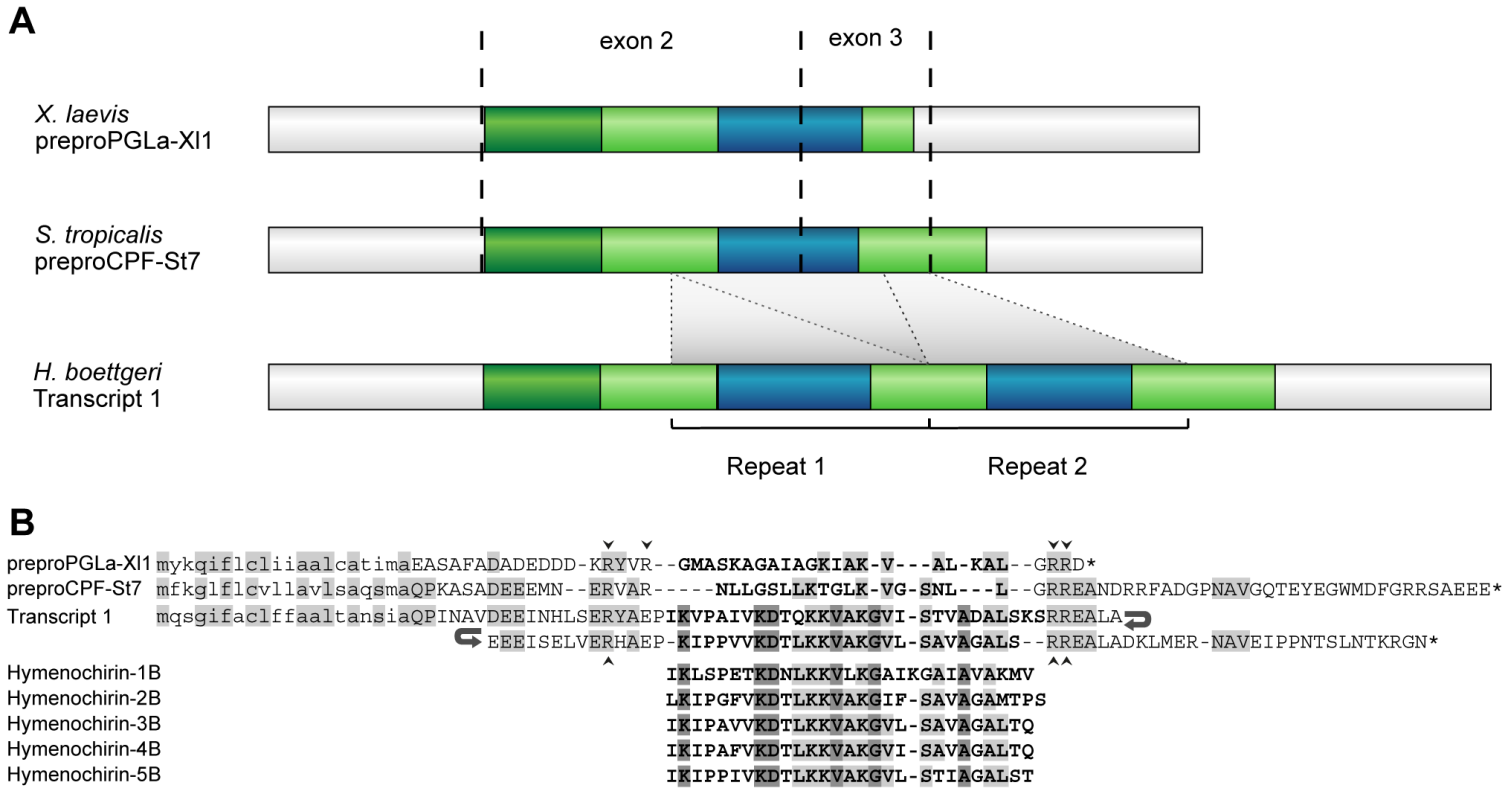
Structure of hymenochirin transcript 1 and comparison to other precursors and AMPs. **A** Structure of hymenochirin transcript 1 (obtained from a breeding skin gland cDNA library of *Hymenochrius boettgeri* males) and of two AMP precursor proteins from *Xenopus laevis* and *Silurana tropicalis*. preproPGLa-Xl1: *X. laevis* PGLa precursor; preproCPF-St7: *S. tropicalis* CPF precursor. Region coloration distinguishes UTR (white), and sequences encoding signal peptide (dark green), spacer (light green) and antimicrobial peptides (blue). Dashed lines indicate known exon boundaries in *Xenopus* and *Silurana* AMP precursors. **B** Comparative alignment of the deduced amino acid sequence of the coding part of hymenochirin transcript 1 with two *X. laevis* and *S. tropicalis* AMP precursors and the previously published hymenochirins [27]. Predicted signal peptides are printed in lower case, antimicrobial peptides are printed in bold. Amino acids shared between the hymenochirin precursor and at least one of the other precursors are indicated in light grey. Residues identical between all hymenochirin peptides but not present in preproPGLa-Xl1 or preproCPF-St7 are labeled in dark grey. Small arrowheads indicate putative cleavage sites.

Randomly sequenced clones of the cDNA library revealed a transcript of 807 base pairs (bp) with an open-reading frame of 432 bp (Figure 1A; transcript 1). BLAST searches indicate that the corresponding protein resembles AMP precursor proteins of *X. laevis* and *S. tropicalis* (Figure 1B). This similarity is most prominent at the N-terminal side of the query sequence, where a predicted signal peptide of 20 AAs is followed by an acidic spacer sequence. In addition, the protein contains two tandem repeats of 138–147 bp (repeat 1 and repeat 2, Figure 1A). Both repeats correspond to the AMP-encoding region of most *X. laevis* and *S. tropicalis* AMP genes which spans part of their exon 2 and exon 3. Similar repeats characterize the magainin and CPF genes in *X. laevis* and arose by tandem duplication of exons 2 and 3 [13], [34]. The *H. boettgeri* repeats are likely to have originated by tandem duplication of the same two exons as well. When translated, the central regions of both repeats show strong sequence similarity to the recently described hymenochirin peptides (Figure 1B), confirming that transcript 1 encodes a hymenochirin precursor protein.

**Figure 2:**
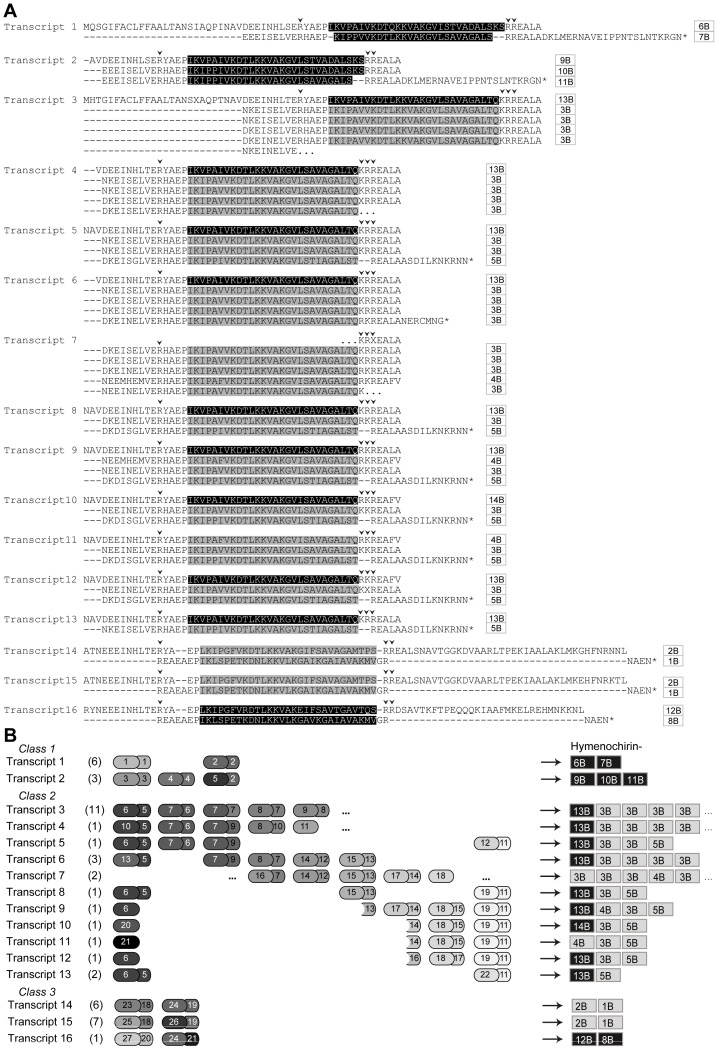
Overview of hymenochirin transcripts: amino acid sequences, structure and encoded peptides. **A** Deduced amino acid sequences of the hymenochirin transcripts. Previously published hymenochirins [27] are marked in grey; predicted novel encoded hymenochirins are marked in black. Small arrowheads indicate putative cleavage sites for the hymenochirins. Names of encoded hymenochirins are indicated on the right. **B** Comparative schematic representation of repeat sequences in the transcripts. The number of cDNA sequences represented by each transcript is indicated between brackets. Each repeat is represented by one larger and one smaller block (repeat sections), corresponding to exons 2 and 3 in *S. tropicalis* and *X. laevis* AMP genes. The numbers in the blocks correspond to unique repeat sections as used in the phylogenetic analyses. Hymenochirins encoded by the corresponding transcripts are indicated on the right; previously published hymenochirins [27] are labeled grey, the novel hymenochirins are labeled black.

Screening of an RNA extract prepared from *H. boettgeri* skin samples using RACE PCR yielded 15 additional transcripts, all containing minimum two tandem repeats (Figure 2A). Based on repeat sequence variation, the transcripts can be grouped into three major repeat classes (Figure 2B). Within each class, sequence variation at the DNA level between tandem repeats in a single transcript and between repeats in different transcripts is often low or even non-existent. Within class 2 (transcripts 3–13), identical repeats seem to be organized in the same relative order in transcripts of different lengths and repeat numbers. This pattern, combined with the matching of single repeats with exons in *X. laevis* and *S. tropicalis* AMP genes, could be explained by alternative splicing among tandemly duplicated exons.

### Hymenochirin Peptide Diversity

**Figure 3:**
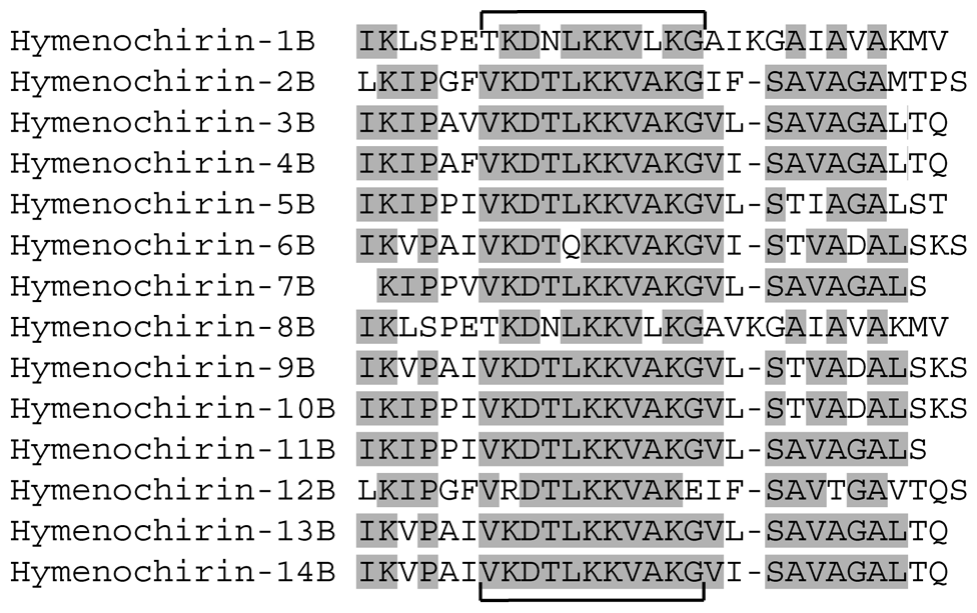
Alignment of all known and predicted hymenochirin peptides. Amino acids shared by more than 50% of the peptides are marked in grey. Brackets delineate a conserved central sequence motif.

Analysis of the 16 transcript sequences indicates that their repeats encode a repertoire of at least 14 different peptides (Figure 2). Besides the five previously described hymenochirins, these include nine new peptides, which we name hymenochirin-6B through hymenochirin-14B (Figure 2, Figure 3). All hymenochirins are 26–29 AA in length and are flanked by arginine-based cleavage sites similar to those of AMPs in other amphibians. There is considerable variation in the number of repeats by which each hymenochirin is encoded. While some peptides are represented only by a single repeat (e.g., hymenochirin-6B), others are encoded by multiple repeats spread across different transcripts (e.g., hymenochirin-13B). Hymenochirin-3B is by far the most frequently encoded peptide, matching 23 repeats in 10 different transcripts (Figure 2A, 2B).

**Figure 4:**
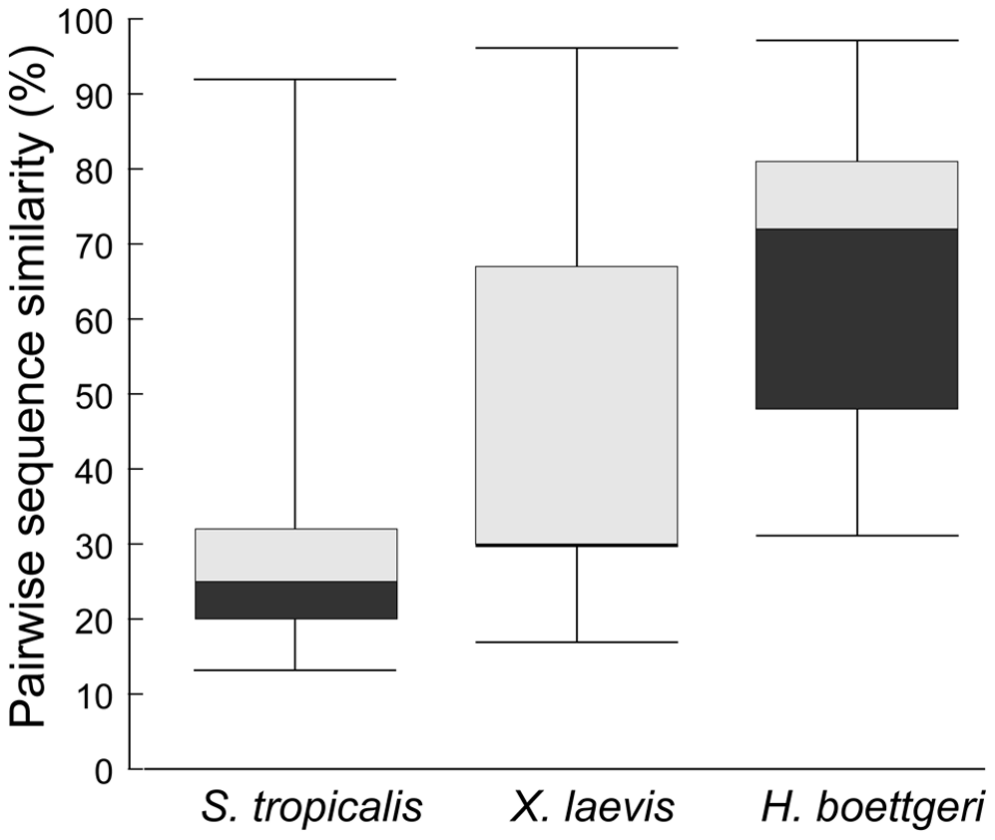
Comparison of pairwise sequence similarities between AMPs of *H. boettgeri*, *S. tropicalis* and *X. laevis*. Box plots comparing the distribution of pairwise sequence similarities (in %) between the 14 hymenochirins of *H. boettgeri* and all known AMP peptides of *S. tropicalis* and *X. laevis* respectively. Boxes indicate median, and 25- and 75- percentiles, and whiskers indicate minimum and maximum values.

The number of different hymenochirins predicted here is comparable to the numbers of known AMPs in other pipid species, like *S. tropicalis* (13 homologous AMPs [13]) and *X. laevis* (12 homologous AMPs). However, sequence diversity is notably lower in *H. boettgeri* compared to the other pipids. Pairwise comparison of the 14 hymenochirins reveals sequence similarities between 31% and 97% (median 72%). In *S. tropicalis* and *X. laevis*, pairwise sequence similarities range between 13% and 92% (median 25%) and between 17% and 96% (median 30%), respectively (Figure 4). The high sequence similarity in hymenochirins is most apparent in the central region of the peptides, where 10 of the 14 peptides share the same 11-AA-long motif (VKDTLKKVAKG), whereas the four others have similar sequences (TKDNLKKVLKG in hymenochirin-1B and -8B, VKDTQKKVAKGV in hymenochirin-6B and VRDTLKKVAKE in hymenochirin-12B) (Figure 3). In addition, the residues that do differ among different hymenochirins are often similar in charge and hydrophobicity.

### Structural and Functional Analysis of New Hymenochirins

**Figure 5:**
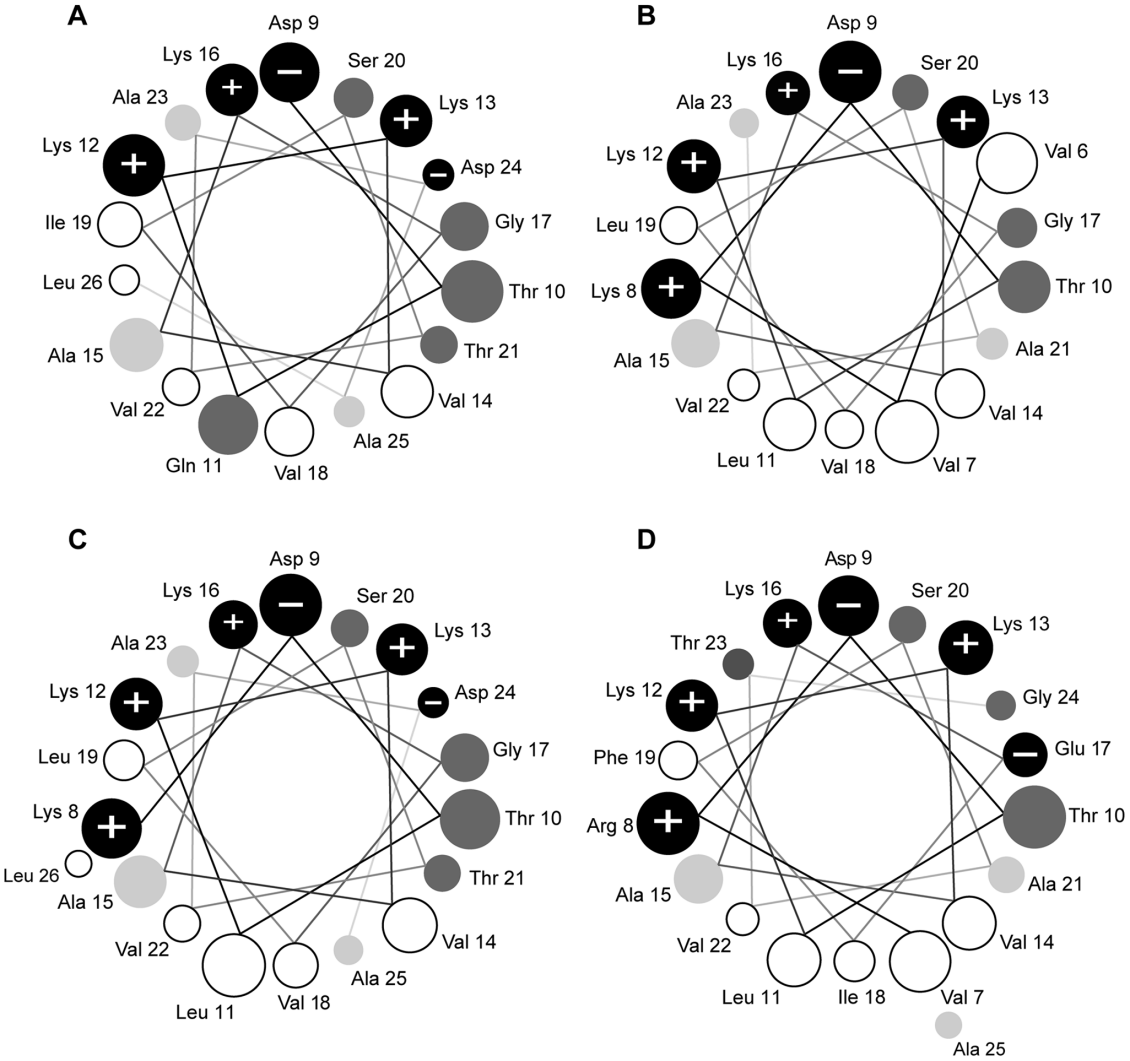
Helical wheel projections of the four peptides that were synthesized and tested for antimicrobial activity. **A** hymenochirin-6B; **B** hymenochirin-7B; **C** hymenochirin-10B; **D** hymenochirin-12B. Only the region of the peptide predicted to have an alpha-helical structure with a confidence level of 5 or more (PSIPRED v3.0 [30]) is shown in the projection. Amino acids are shaded according to the Combined Consensus Scale [31] with hydrophobic residues in white, nearly neutral residues in light grey, polar residues in dark grey, charged residues in black with+or - sign indicating charge. Helical wheels are adapted from http://cti.itc.virginia.edu/~cmg/Demo/wheel/wheelApp.html. Circle sizes indicate the relative distance from the N-terminal end of the peptide; smaller circles are further away.

All peptides newly predicted from the *H. boettgeri* transcripts share the typical features of AMPs. First, secondary structure predictions suggest that all of them have the potential to form an alpha-helix, and the predicted degree of helicity ranges between 77% (hymenochirin-11B) and 82% (hymenochirin-8B and -9B) of the total peptide length (Table 1). Second, all of them are cationic, with net charges ranging between +3 (hymenochirin-12B) and +5 (hymenochirin-8B) at a pH of 7.0. Third, they show an alternated sequence of hydrophilic/cationic and hydrophobic AAs, which in an alpha-helical configuration results in two opposite faces along the helical axis, as visualized by helical wheel projections (Figure 5).

**Table 1:** Structural properties of the novel hymenochirin peptides predicted from the *H. boettgeri* transcripts.

Peptide	Averagemass (mol)	Chargeat pH7	Mean hydrophobicmoment	Meanhydrophobicity	Helicity
Hymenochirin-6B	2995.575	+4	1.83	−1.83	79%
Hymenochirin-7B	2590.168	+4	2.56	−0.76	80%
Hymenochirin-8B	3049.778	+5	2.6	−1.48	82%
Hymenochirin-9B	2980.604	+4	2.38	−1.25	82%
Hymenochirin-10B	3020.668	+4	2.38	−1.06	79%
Hymenochirin-11B	2717.354	+4	2.53	−0.3	77%
Hymenochirin-12B	3104.661	+3	2.43	−0.94	79%
Hymenochirin-13B	2819.447	+4	2.41	−0.68	82%
Hymenochirin-14B	2819.447	+4	2.41	−0.72	82%

Average mass was calculated with http://www.peptidesynthetics.co.uk/tools. Hydrophobicity was calculated following the Combined Consensus Scale [31] as implemented in HydroMCalc. The mean hydrophobic moment is the vectorial sum of all the hydrophobicity indices, divided by the number of residues. The mean hydrophobicity is the total hydrophobicity (sum of all residue hydrophobicity indices) divided by the number of residues. Helicity was calculated with PSIPRED v3.0 [30].

Four of the newly predicted peptides, selected based on their high variation in primary structure, were *de novo* synthesized and submitted to minimum inhibitory concentration (MIC) assays and hemolysis tests (Table 2). One of these peptides, hymenochirin-6B, seems to be devoid of any antimicrobial activity (all MIC values >512 µM). On the contrary, hymenochirin-7B, -10B and -12B show moderate to weak activity against the gram-positive species *S. aureus* (128–512 µM) and very strong activity against the gram-negative bacteria *E. coli* (<1–2 µM) and *P. aeruginosa* PAO1 (4–16 µM). The same low MIC values were obtained when these three peptides were tested against the multiresistant *P. aeruginosa* strain PA7 [33]. In contrast, the potency of these three peptides to induce lysis of red blood cells is very low: while hymenochirin-12B induced 50% hemolysis (HC_50_) at a concentration of 256 µM, hymenochirin-7B and -10B both have HC_50_ values higher than 512 µM. Moreover, the hemolytic activity of hymenochirin-7B and hymenochirin-10B approximates zero % at concentrations ≤ 64 µM and ≤ 32 µM, respectively.

**Table 2:** Minimum inhibitory concentrations (MIC) of synthetic hymenochirins against bacteria and concentration causing at least 50% hemolysis of human red blood cells (HC50).

	MIC				
Tested peptide	*E. coli*	*S. aureus*	*P. aeruginosa* PAO1	*P. aeruginosa* PA7	HC50
Hymenochirin-6B	>512 µM	>512 µM	>512 µM	>512 µM	ND
Hymenochirin-7B	2 µM	128–256 µM	16 µM	16 µM	>512 µM
Hymenochirin-10B	<1 µM	256–512 µM	4–8 µM	8 µM	>512 µM
Hymenochirin-12B	<1 µM	128 µM	4 µM	4 µM	256 µM

ND: not determined.

### Evolution of Hymenochirins

**Figure 6:**
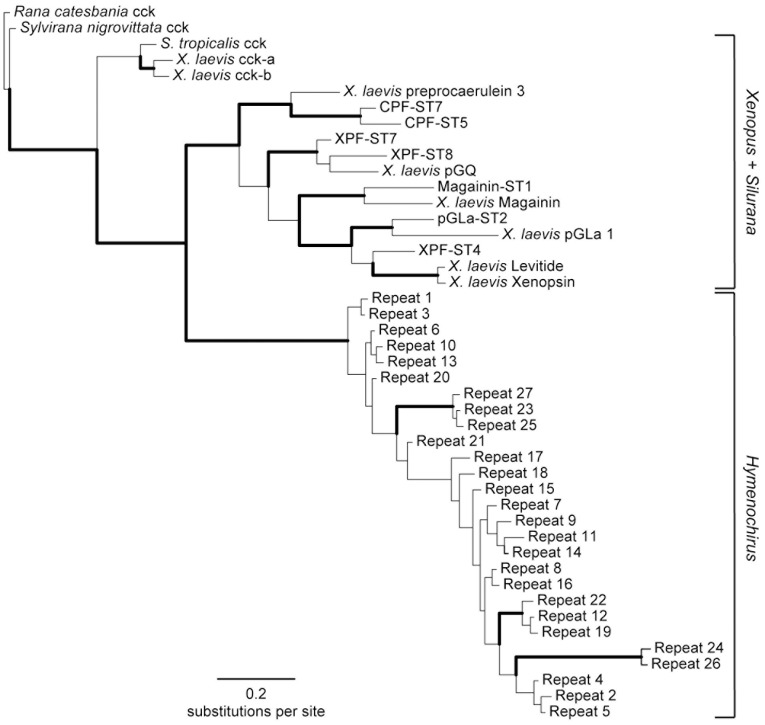
Figure 6. Phylogenetic relationships among pipid AMPs (exon 2). Phylogenetic relationships as inferred by Bayesian analysis of a data set consisting of exon 2 of amphibian cck genes and AMP genes in *X. laevis* and *S. tropicalis*, aligned with the corresponding repeat sections of the hymenochirin precursor proteins. Repeat numbers correspond to those in Figure 2. The depicted tree represents the Bayesian consensus phylogram rooted with cck genes. Branches are shown in bold if Bayesian posterior probability is above 0.95 and RAxML bootstrap is more than 75%.

Phylogenetic analyses elucidate the evolutionary position of hymenochirins with respect to other pipid AMPs (Figure 6). Both ML bootstrapping and Bayesian analyses of data set 1 (anterior repeat section matching exon 2 of cck and other pipid AMP genes) deliver strong support for a clade composed of all hymenochirin repeats (ML bootstrap support (MLBS) = 100%; Bayesian posterior probability (BPP) = 1.0) to the exclusion of all remaining AMPs (Figure 6). The *S. tropicalis* and *X. laevis* precursors constitute a clade as well (MLBS = 94%; BPP = 0.97), implying a basal divergence between the hymenochirins and remaining pipid AMPs. These results are consistent with the prediction that the structural dissimilarity of hymenochirins with respect to other pipid AMPs reflect the early divergence of *Hymenochirus* and the *Silurana*+*Xenopus* lineage within Pipidae [27].

Notably, the branches in the clade of *Xenopus* and *Silurana* AMP genes are much longer than the branches in the hymenochirin clade, in accordance with the low sequence variation among hymenochirins compared to other AMPs. This contrast may indicate that hymenochirins, unlike the *Silurana* and *Xenopus* AMPs [13] may have experienced little diversifying selection during their evolution, and that purifying selection even constrained their divergence. Selection analyses using any of the three likelihood methods (see Materials and Methods) indicate an apparent lack of significant diversifying selection. Only the REL method, known to be more liberal than e.g. SLAC or FEL (i.e. more likely to predict false positives [40]) identified a single codon site in the peptide-encoding region as subject to significant diversifying selection (see Table 3), but this in only eight of the 20 analyzed trees. Second, all methods indicate that purifying selection acted both on cleavage sites and on hymenochirin peptides, although the number of subjected codon sites estimated varies considerably among methods. Only three codon sites (encoding Arg and Glu in the N-terminal cleavage site and Pro at position 4 in most hymenochirins) were consistently identified by all three methods as subject to significant purifying selection. Three additional sites (encoding Ala in the N-terminal cleavage site, Asp at position 9, and Lys at position 16 in the hymenochirins) were identified by two methods (FEL and REL). Three other sites (encoding Lys at positions 3 and 13, and Val at position 14) are constant across repeats, but were nevertheless identified by REL as subject to purifying selection.

**Table 3:** Number of codon sites predicted to evolve under diversifying or purifying selection according to three different methods.

		REL^a^		FEL^b^		SLAC^b^	
Sequence encoding:	Type of selection	Consensus^c^	(min–max)^d^	Consensus^c^	(min–max)^d^	Consensus^c^	(min–max)^d^
Cleavage sites	diversifying	0	(0–0)	0	(0–0)	0	(0–0)
	purifying	4	(4–4)	3	(3–4)	2	(1–2)
Hymenochirin peptide	diversifying	1	(0–1)	0	(0–0)	0	(0–0)
	purifying	11	(6–12)	4	(3–5)	2	(0–3)

a Using a Bayes factor of 50.

b Using a 0.05 significance level.

c calculated on the Bayesian consensus trees.

d minimum and maximum number of codon sites calculated on 20 randomly selected trees from the Baysesian posterior tree set.

## Discussion

We here report on the potential of the dwarf clawed frog *Hymenochirus boettgeri* to produce at least 14 different hymenochirins, thereby almost tripling the number of known AMPs of this species. At least three of them show promising activity against pathogens that pose an acute threat to clinical healthcare. Due to their efflux systems, antibiotic modifying enzymes, and ability to form biofilms, *P. aeruginosa* strains, like PAO1 (one of the strains tested in this study), are resistant to conventional penicillins and cephems [42]. The strain PA7 is additionally resistant to third generation cephalosporins, monobactams, fluoroquinolones, piperacillin, carbenicillin, levofloxacin, chloramphenicol [33] and to aminoglycoside antibiotics such as gentamycin and tobramycin [43]. The similarly high sensitivity of PAO1 and PA7 to multiple hymenochirins, combined with their low hemolytic activity suggests that these peptides may be useful lead compounds for the development of a new class of antibiotics against this multi-resistant pathogen.

One of the four peptides tested, hymenochirin-6B, shows at least a 1000-fold decrease in antimicrobial activity compared to the other three. Pairwise comparison of AA sequences shows that it is very similar to hymenochirin-10B, from which it differs by four AAs. In three cases (Val instead of Ile at position 3, Ala instead of Pro at position 5, Ile instead of Leu at position 19) these different AAs are physiochemically relatively similar, bearing little variation in the overall charge, hydrophobicity or amphipathicity of the peptides. At position 11 however, hymenochirin-6B contains a strongly hydrophilic Gln residue instead of a strongly hydrophobic Leu. This Gln residue not only reduces the peptide’s overall hydrophobicity, but because position 11 is situated at the hydrophobic side of the predicted helix, it is likely to affect the peptide’s amphipathic nature (Figure 3, compare A to C). The latter is confirmed by the difference in the calculated hydrophobic moments of hymenochirin-6B and hymenochirin-10B (1.83 vs. 2.38 following the hydrophobicity scale of Tossi et al. [31]). The observation of similarly weak AMPs in other pipid species recently fuelled the idea that such peptides may have evolved an alternative function (neofunctionalization) [16].

Our cDNA data provide insights in the transcript diversity *H. boettgeri* is able to produce, but the exact number of underlying genes remains unknown. Based on the encoded peptides, we distinguished three transcript classes (Figure 2B), which may represent diverged gene lineages. A conspicuous feature is the occurrence of identical repeat units, both in different transcripts of the same class, and within the same transcript. The arrangement of identical repeats in different transcripts may indicate that the latter arose by alternative splicing of a single gene that underwent exon duplication. However, the presence of identical repeats in the same transcript cannot be explained by the use of a single exon and implies that identical exons occur in a *cis*-arrangement in the frog’s genome. A similar pattern is observed in some of the related AMP genes of *X. laevis*: caerulein and magainin genes contain several tandem-duplicated exons encoding a battery of (nearly) identical peptides. Such repeats add little structural diversity to a frog’s peptide arsenal but instead may provide a means to control the overall AMP production through alternative splicing or differential peptide cleavage [44].

**Figure 7:**
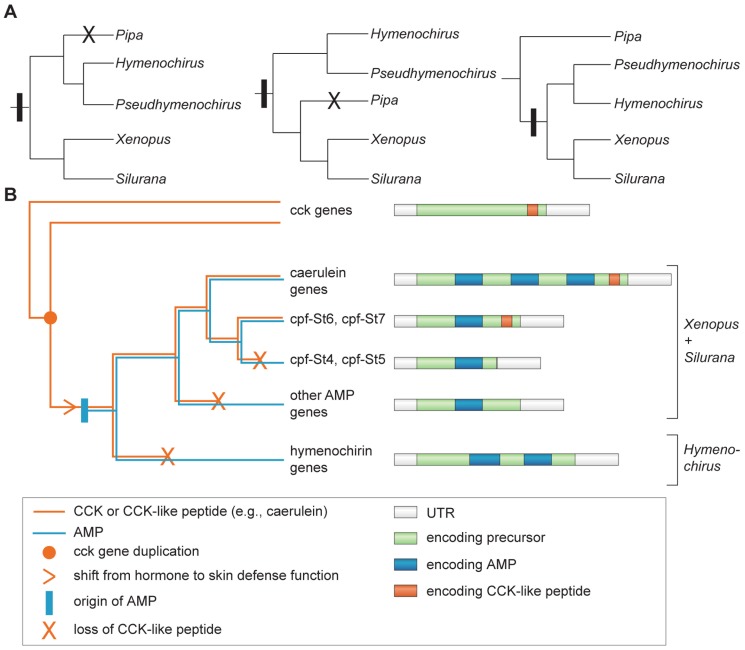
Figure 7. Origin and loss of AMPs and CCK-like peptide in the family Pipidae. **A** The three recently postulated phylogenetic hypotheses for the family Pipidae and their implications for the origin (vertical bars) and loss (crosses) of an AMP gene repertoire. **B** Summarized gene tree illustrating the evolution of the pipid AMP gene family.

Our phylogenetic analyses support the hypothesis that *Hymenochirus* AMPs diversified in parallel to those in *Xenopus* and *Silurana*. In addition, this independent diversification proceeded under different evolutionary regimes. Amphibian AMPs typically show large intraspecific variation, either as a result of diversifying selection in adaptation to a diverse spectrum of infectious microorganisms [9], [10], or because AMPs lack a specific target (like a hormone receptor) and do not require the preservation of a specific primary structure [6]. Hymenochirins however, show relatively little structural variation compared to the AMPs of other amphibian species, despite being comparable in number. It is unlikely that this low variation is caused by the study of only six (potentially closely related) individuals. First, the peptides predicted in this study include all peptides previously identified by peptidome analysis of eight *H. boettgeri* specimens from populations in Cameroon and the Democratic Republic of the Congo [27], thereby encompassing the peptide repertoires of geographically distant populations. Second, the pairwise structural variation among predicted hymenochirins of six *H. boettgeri* individuals is lower than among the 13 AMPs predicted from the *S. tropicalis* genome sequence, which represents only one individual [13]. Finally, several recent skin peptidome and transcriptome studies identified a much larger structural diversity in host-defense peptides, despite being conducted on only one or two individual frogs [7], [13], [21], [23], [25]. The most extreme case of structural diversity was reported for the ranid species *Odorrana grahami*, where a single frog was found to produce 107 different peptides that were classified in 30 structural families [7]. A recent peptidome study on the skin secretions of 10 individuals of the species *Pseudhymenochirus merlini* (the only species of the pipid genus *Pseudhymenochirus*) showed a similarly low level of structural variation among its 13 host-defense peptides [45]. Our selection analyses indicate that purifying selection at least partially clarifies the low sequence variation among hymenochirins. However, additional explanations, other than purifying selection at the peptide level, may underlie the overall low sequence variation among transcript repeats. First, nearly identical repeat units could simply represent recently duplicated exon(s). In this case, these exons merely lacked the time to diverge. Second, low variation at the DNA level could reflect an overall low substitution rate, either because the repeat-encoding regions are ‘protected’ against mutation by epigenetic features (e.g., DNA methylation [46]) or efficient DNA repair systems [47] or because the DNA sequences *themselves* are subject to purifying selection. Selection to maintain translational efficiency, splicing, RNA secondary structure, and specific nucleosome positioning has been shown to operate on synonymous sites in coding sequences [48], [49]. Such purifying selection at the DNA level has recently been reported in diverse eukaryotes [49], [50] and has been postulated to account for the conserved gene regions encoding the signal peptides of scorpion toxin precursors [51]. Third, low genetic variation among repeats could be maintained by the concerted evolution of duplicated genes or exons [52], [53], [54]. The exchange of sequences between paralogous genes/exons through gene conversion or unequal crossing-over may set back any genetic divergence that arose after duplication, leading to the homogenization of corresponding peptide sequences. We anticipate that similar genetic mechanisms may explain the similarly low variation in *P. merlini*. The tree recovered by our phylogenetic analyses of transcript repeats is consistent with the phylogeny of pipid genera as supported by both morphological and molecular analyses, with an early divergence between *Hymenochirus* and a lineage composed of *Silurana* and *Xenopus* [55], [56], [57], [58], [59], [60], [61], [62], [63], [64], [65]. However, evolutionary relationships among these genera have been controversial with respect to a fourth genus, *Pipa*. Phylogenetic studies of the past 20 years have variously supported: (*i*), the basal divergence between a *Pipa*+*Hymenochirus* lineage (Pipinae) and *Silurana*+*Xenopus* lineage (Xenopodinae) [56], [57], [65], [66], [67] (Figure 7A, left), (*ii*), the basal divergence of *Hymenochirus*, followed by the divergence of *Pipa* [59] from *Silurana*+*Xenopus* (Figure 7A, middle), and (*iii*), a basal divergence of *Pipa*, followed by the divergence of *Hymenochirus* from *Silurana*+*Xenopus* [58], [60], [61], [62], [63], [64] (Figure 7A, right). These three phylogenetic hypotheses implicate alternative scenarios of AMP arsenal evolution in Pipidae. Peptidome screening of the species *Pipa pipa* did not reveal any AMP [68], [69]. Similarly, application of the same cDNA techniques on *Pipa parva* as reported here for *H. boettgeri*, did not reveal transcripts encoding homologous peptide precursors (unpublished data). AMP production could have been overlooked by either approach, but under the first two phylogenetic hypotheses, the absence of AMPs in *Pipa* would require the origin of an AMP gene in a common ancestor of all extant Pipidae, followed by secondary loss in *Pipa* (Figure 7A, left and center). Instead, the basal divergence of *Pipa* would yield a more parsimonious scenario, requiring the origin of an ancestral AMP gene in a common ancestor of *Hymenochirus*, *Xenopus* and *Silurana* without any secondary loss (Figure 7A, right). The recently identified host-defense peptides in *P. merlini* are very similar to the hymenochirins of *H. boettgeri* [45]. This is in line with a sister-clade relationship between the genera *Hymenochirus* and *Pseudhymenochirus* as opposed to *Xenopus*+*Silurana*. These findings support the scenario of a basal divergence of *Pipa* and the origin of an ancestral host-defense gene in the common ancestor of all other Pipidae (Figure 7A, right).

Although the hymenochirin genes, and their orthologues in *Silurana* and *Xenopus*, descended from a hormone gene, their AMPs are functionally unrelated to the CCK hormone, marking a clear-cut neofunctionalization event. A summarized gene tree (Figure 7B) allows us to reconstruct the key determinant steps involved in the functional evolution of the AMP gene family in pipid frogs. It entails an ancestral gene duplication of the cck gene followed by (1) the evolutionary transition of one of the duplicates from a CCK hormone function to a CCK-like defense function, and (2), the origin of a second, antimicrobial function (by cleavage of a second peptide from the same precursor). We recently postulated that a CCK-like defense function arose by a shift in gene expression to the skin, without the loss of the capacity to bind CCK receptors [13]. This hypothesis relies on the fact that some genes in *X. laevis*, besides encoding AMPs, retained the capacity to produce a CCK-like peptide, known as caerulein. Caerulein is capable to bind CCK receptors in other vertebrates, causing acute pancreatitis, vomiting, diarrhea, hypotension, and inhibition of exploratory and feeding behavior [70]. Caerulein-related peptides have been found in other *Xenopus* species [22], and seem to be encoded by two AMP genes of *S. tropicalis* (cpf-St6 and cpf-St7; Figure 7B [13], [34]). The absence of CCK-like peptides in other *Xenopus* and *Silurana* AMP genes implies two parallel secondary losses (Figure 7B; [13]). None of the currently identified hymenochirin transcripts encodes a CCK-like peptide, which, in light of the gene tree, marks a third loss.

In the absence of known precursor sequences for *P. merlini*, it is impossible to conclude whether loss of the CCK-like defense function occurred before the divergence of *Hymenochirus* and *Pseudhymenochirus*, or whether it occurred later in the *Hymenochirus* lineage. So far however, peptidome analyses did not reveal CCK-like peptides in either genus. After loss of the primary CCK-like defense function, selection to retain the secondarily derived host-defense peptide function may have rescued the hymenochirin genes (and potentially related *P. merlini* genes) from evolutionary degradation and pseudogenization. Hence, the evolution of secondary peptides may be an important way by which defense genes in expanding gene families may recurrently undergo parallel functional shifts.

## Supporting Information

Checklist S1 **The ARRIVE Guidelines Checklist.**
(DOC)
